# Lnc-GULP1–2:1 affects granulosa cell proliferation by regulating COL3A1 expression and localization

**DOI:** 10.1186/s13048-021-00769-1

**Published:** 2021-01-20

**Authors:** Guidong Yao, Yue Kong, Guang Yang, Deqi Kong, Yijiang Xu, Jiahuan He, Ziwen Xu, Yucheng Bai, Huiying Fan, Qina He, Yingpu Sun

**Affiliations:** 1 Center for Reproductive Medicine, the First Affiliated Hospital of Zhengzhou University, Zhengzhou, China; 2 Henan Key Laboratory of Reproduction and Genetics, the First Affiliated Hospital of Zhengzhou University, Zhengzhou, 450052 China

**Keywords:** lncRNA, COL3A1, Ovarian follicular development, Granulosa cell, Cell proliferation

## Abstract

**Backgrounds:**

Long non-coding RNA is a novel group of non-protein coding transcripts over 200 nt in length. Recent studies have found that they are widely involved in many pathological and physiological processes. In our previous study, we found that lnc-GULP1–2:1 was significantly down-regulated in the ovarian cortical tissue of patients with primary ovarian insufficiency and predicted that lnc-GULP1–2:1 has a regulatory effect on COL3A1.

**Results:**

In this study, we found that lnc-GULP1–2:1 was mainly localized in the cytoplasm of luteinized granulosa cells. The expression of lnc-GULP1–2:1 was lower in patients with diminished ovarian reserve but substantially elevated in patients with polycystic ovary syndrome. Overexpression of lnc-GULP1–2:1 in KGN cells significantly inhibited cell proliferation, likely through cell cycle related genes CCND2 and p16. Moreover, lnc-GULP1–2:1 expression was positively correlated with the level of COL3A in luteinized granulosa cells from patients with different ovarian functions as well as in multiple cell lines. Overexpression of lnc-GULP1–2:1 in KGN cells promoted the expression of COL3A1 and its translocation into the nucleus. Consistently, silencing COL3A1 in KGN cells also significantly inhibited cell proliferation.

**Conclusions:**

Lnc-GULP1–2:1 affects the proliferation of granulosa cells by regulating the expression and localization of COL3A1 protein, and may participate in the regulation of ovarian follicle development. This study will provide new insight into molecular mechanisms underlying ovarian follicular development, which will help generate novel diagnostic and therapeutic strategies for diseases related to ovarian follicular development disorders.

**Supplementary Information:**

The online version contains supplementary material available at 10.1186/s13048-021-00769-1.

## Introduction

Long non-coding RNA (lncRNA) is a kind of non-encoding RNA molecules with a transcript length of more than 200 nt [1–3]. It should be noted that lncRNA is a generic term that encompasses different classes of RNA transcripts, including enhancer RNA, snoRNA hosts, intergenic transcripts, and sense or antisense transcripts that overlap other transcripts [4]. Unlike miRNAs or proteins, the function of most lncRNAs cannot be inferred from the sequence or structure [5]. At present, the major described cellular functions of lncRNA are chromatin modification, transcriptional and post-transcriptional gene expression regulation [5, 6].

LncRNAs are involved in a variety of physiological and pathological processes, including proliferation, differentiation, developpment, apoptosis and carcinogenesis [7–9]. For example, overexpression of lncRNA-Amhr2 in mouse granulosa cells results in decreased mRNA levels of Amhr2, and activation of lncRNA-Amhr2 increases Amhr2 promoter activity [8]. Furthermore, the expression of lncRNAs and their regulation of target genes are highly tissue-specific. Of note, most of the studies so far have been focused on the identification of new lncRNAs, but failed to elucidate the functions of specific lncRNAs, especially in human ovarian granulosa cells.

Primary ovarian insufficiency (POI) refers to the loss of ovarian function before the age of 40, accompanied by amenorrhea, hypergonadotropism, and hypoestrogenism [10, 11]. In most cases, the specific mechanism leading to premature depletion of the primordial follicle pool is unclear. Genetic disorders, autoimmune diseases, tuberculosis of the genital tract, smoking, ovarian surgery, radiation and/or chemotherapy are potential causes of POI [11]. Although many genetic mutations and toxic agents are destructive to oocytes, the role of oocyte destruction in the pathogenesis of POI remains to be explained.

During follicular development, granulosa cells surround the oocyte, and subsequently differentiate into either mural granulosa cells or cumulus granulosa cells [12]. The physiological function of granulosa cells depends on both paracrine and autocrine cytokines in the ovarian microenvironment as well as reproductive hormones in peripheral blood [13, 14]. Oocytes and granulosa cells grow and develop in a highly coordinated, interdependent manner [15–18]. Granulosa cells provide nutrient and maturation-related factors to oocytes, ensuring the development and maturation of oocytes [19]. Granulosa cells apoptosis is a physiological phenomenon in follicles. The loss of granulosa cells and the destruction of cell-cell communication may cause lack of nutrients and survival factors in pre-ovulatory oocytes, thus leading to oocyte apoptosis and follicular atresia [20], and eventually resulting in the depletion of follicles [21]. The gradual loss of granulosa cells is a key factor in antral follicle atresia. Therefore, granulosa cells dysfunction is a critical step leading to abnormal follicular development [22, 23].

In our previous study, next generation sequencing revealed that lnc-GULP1–2:1 and collagen type III alpha 1 chain (COL3A1) were significantly down-regulated in ovarian cortical tissues form patients with POI compared with those in normal controls [24]. Co-expression network analysis showed that COL3A1 was the *cis* regulated transcript of lnc-GULP1–2:1 [4, 25]. Lnc-GULP1–2:1 is 628 bp in length (the detailed information of lnc-GULP1–2:1 is provided in supplementary material), and it overlaps with the 5′ end of COL3A1 gene [24]. Furthermore, 1-378 bps of lnc-GULP1–2:1 overlaps with exon 1 and 2 of COL3A1 gene; 379-612 bps of lnc-GULP1–2:1 completely overlaps with exon 2 and the intron between exon 2 and 3 of COL3A1 gene; 613-628 bps of lnc-GULP1–2:1 partially overlaps with exon 3 of COL3A1 gene. Through sequence analysis and comparison, we believe that lnc-GULP1–2:1 may be the alternative splicing product of COL3A1 gene.

Therefore, we speculate that lnc-GULP1–2:1 may affect the function of granulosa cells by regulating COL3A1 gene expression. In view of this, this study explored the expression level of lnc-GULP1–2:1 in granulosa cells of patients with different ovarian functions and its influence on granulosa cell proliferation and cell cycle regulation, and further investigated the role of lnc-GULP1–2:1 in the modulation of COL3A1 expression.

## Materials and methods

### Sample collection

A total of 22 patients were enrolled, including 7 patients in the normal group, 6 patients in the diminished ovarian reserve (DOR) group, and 9 patients in the polycystic ovary syndrome (PCOS) group. Normal group were patients younger than 35 years old with regular menstrual cycles; DOR group were patients affected by secondary infertility older than 40 years [24]; PCOS patients were younger than 35 years old and were diagnosed according to the Rotterdam 2003 criteria [26], which require the presence of two of the following three criteria: ultrasound demonstration of polycystic ovaries, chronic anovulation and hyperandrogenism. All the enrolled patients underwent follicular aspiration for the first time and had no history of ovarian surgery.

### Primary luteinized granulosa cell isolation and cell culture

Primary human granulosa cells were extracted and purified from the follicular fluid aspirates utilizing density centrifugation (Lymphocyte Separation Medium, LTS1077, Tianjin, China). Cell sedimentation was then washed twice with ice-cold phosphate-buffered saline (PBS), and resuspended in DMEM/F12 (Gibco, Life Technologies, Carlsbad, CA, USA) containing 10% fetal bovine serum (FBS, Gibco) and 50 U/mL penicillin-streptomycin (Gibco). KGN, a human granulosa-like tumor cell line, was gifted from Prof. Fei Sun (University of Nantong, China). The lentivirus KGN cell line stably overexpressing lnc-GULP1–2:1 (Lv-lnc-GULP1–2:1) and or its control (Lv-EGFP) was constructed using the similar method as in previous studies [24]. KGN, MDA-MB-231 cells were cultured in DMEM/F12 medium (Gibco). 293 T, BeWo and HTR-8/SVneo cell lines were cultured in high-glucose DMEM containing glutamax1 (Invitrogen, Paisley, UK). OVCAR3 cells were cultured in Low Glucose DMEM and SKOV3 cells were cultured in RPMI1640 containing glutamax1 (Invitrogen). U87 MG and Hep G2 cells were cultured in MEM (Gibco). JAR was cultured in RPMI-1640 medium (Invitrogen). All the cell lines used in this study have been verified by short tandem repeat (STR) analysis for authenticity, and were routinely cultured at 37 °C in an atmosphere of 5% CO2 in compressed air at high humidity and all media were supplemented with 10% fetal bovine serum (FBS, Life Technologies) and 50 U/mL penicillin-streptomycin (Life Technologies).

### Quantitative RT-PCR

Total RNA was extracted by using Trizol reagent (Life technologies, Inc., Gaithersburg, MD, USA). Total RNA (approximately 800 ng) was reverse transcribed into cDNA by using the iScript™ cDNA Synthesis Kit (Bio-Rad, CA, USA). Briefly, the 20 μl RT reactions (0.8 μg RNA, 5 μl iScript reaction Mix, 1 μl iScript Reverse Transcriptase and ddH_2_O) was incubated for 5 min at 25 °C and 20 min at 46 °C, incubated for 1 min at 95 °C and then maintained at 4 °C. For real-time PCR, all reactions were performed in triplicate with iTaq™ Universal SYBR® Green Supermix (Bio-Rad) under the following conditions: 30s at 95 °C for initial denaturation, followed by 40 cycles of segments of 95 °C for 3 s and 60 °C for 30s in 7500 Fast Real-Time PCR System (Applied Biosystems, Foster City, CA, USA). Glyceraldehyde-3-phosphate dehydrogenase (GAPDH) was used as an internal reference gene. Gene expression was calculated by using the method of 2^−△△Ct^, △Ct = △Ct_target_ − △Ct_reference_, −△△Ct = − (△Ct_sample_ − △Ct_control_). The sequences of primers for real-time PCR are listed in Supplementary Table 1.

### Cell proliferation assays

Cells were inoculated at a density of 1 × 10^4^ cells in 96-well plates, and cultured for 24 h, followed by corresponding treatment. Cell proliferation was measured by using Cell Counting Kit-8 (Boster, Wuhan, China) at 0, 24, 48 and 72 h after treatment. At the indicated time, fresh 100 μl DMEM/F12 plus 10 μl CCK-8 medium was added to each well, incubated for 2 h in cell incubator. OD value was measured at 450 nm using spectrophotometer (Thermo Fisher, Vantaa, Finland). Each experiment was independently repeated 3 times, and each treatment had 6 replicate wells in each group.

### Western blot

Total Protein Extraction Kit (Sangon Biotech, Shanghai, China) was used for total protein extraction according to production instructions. Protein concentrations were measured by using Dye Reagent (Bio-Rad) with Quick Start™ Bovine Serum Albumin (Bio-Rad) as standard. Samples were then boiled in protein loading buffer (Boster) at 100 °C for 10 min, and equal amounts of protein (40 μg) were loaded into the wells of the SDS-PAGE gel. Following by electrophoresis according to standard procedures, the separated proteins were transferred to polyvinylidene fluoride (PVDF) membranes (Bio-Rad) in a wet transfer system (Bio-Rad). Membranes were blocked for 1 h at room temperature in 5% non-fat dry milk in tris-buffered saline supplemented with 0.1% Tween-20 (TBST). After blocking, membranes were incubated with primary antibodies (anti-COL3A1 mouse monoclonal antibody, 1:100, Santa Cruz, Oregon, USA; anti-GAPDH mouse monoclonal antibody, 1:3000, CMCTAG, Milwaukee, USA) in blocking buffer at 4 °C overnight. Then the PVDF membranes were washed 3 times (10 min each) with TBST at room temperature and incubated with secondary antibody (goat polyclonal secondary antibody to mouse IgG-H&L (HRP), 1:5000, Abcam, Cambridge, USA) for 1 h. Finally, immunoreactive bands were detected by using enhanced chemiluminescent substrate in a ChemiDoc MP imaging system (Bio-Rad).

### Immunofluorescence

Lentivirus-stabilized KGN cell lines, which stably overexpressing lnc-GULP1–2:1 (Lv-lnc-GULP1–2:1) and its control (Lv-EGFP), were re-inoculated on glass coverslips pre-coated with poly-lysine and cultured for 24 h in an atmosphere of 5% CO_2_ at high humidity. Cells were treated with 4% paraformaldehyde for 20 min, then gently washed with phosphate buffered saline (PBS) for 3 × 3 min. Next, cells were permeabilized with 0.5% Triton X-100 in PBS for 20 min and blocked by using 5% bovine serum albumin (BSA) in TBST for 30 min at room temperature. After blocking, cells were incubated with COL3A1 antibody (1:100, Santa Cruz) or Ki-67 antibody (1:1000, Cell signaling technology, Beverly, USA) at 4 °C overnight; PBS was used as a negative control. Then cells were washed 3 × 3 min with PBS at room temperature and incubated with Alexa Fluor 594-AffiniPure Goat Anti-Rabbit IgG (1:600, Jackson, Pennsylvania, USA) for 30 min. After washing with PBS, the nuclei were stained with 4′, 6-diamidino-2-phenylindole (DAPI) (5 μg/ml). Then the coverslips were washed again with PBS 4 × 5 min. Finally, the coverslips were mounted with anti-fade Mounting Medium (Beyotime Biotechnology, Shanghai, China), and the image was observed and collected under a laser scanning microscope (Carl Zeiss, Oberkochen, Germany).

### Fluorescence in situ hybridization

Cells were re-plated on poly-lysine-pre-coated glass coverslips and cultured for 24 h in an atmosphere of 5% CO_2_ at high humidity, then treated with 4% paraformaldehyde for 20 min. After washing the coverslips, cells were digested by 20 μg/ml proteinase K (Servicebio, Wuhan, China) for 5 min. Then the coverslips were washed 3 × 3 min with PBS at room temperature and incubated with prehybridization solution for 1 h at 37 °C. Next, the prehybridization solution was discarded, and a hybridization solution (Servicebio) containing lnc-GULP1–2:1 probe (5′-DIG-CATGG CTATTTGATGAACATGACTTT-DIG-3′) or nonsense control probe (5′-DIG GTGTAACACGTCTATACGCCCA-3′) (Genepharma, Shanghai, China) at a concentration of 8 ng/μl, was added dropwise, and hybridization was carried out at 37 °C overnight. Hybridization solution then was washed away. The coverslips were placed in 5% BSA in PBS (Servicebio) for 30 min. The blocking solution was discarded, anti-DIG-cy3 (Jackson, Pennsylvania, USA) was added dropwise, incubated at 37 °C for 50 min, and then washed 3 × 5 min with PBS. The DAPI staining solution was added to the coverslips, incubated for 8 min in the dark, and the anti-fade Mounting Medium (Servicebio) was added after washing. Finally, the slides were observed under a fluorescence microscope (NIKON ECLIPSE CI, JAPAN).

### RNA interference

For gene knockdown analysis, COL3A1 specific siRNA (sc-43,062, Santa Cruz) was used to silence the COL3A1 gene, and siRNA control (Silencer™ Select Negative Control, 4,390,843, Invitrogen) was used as a silence control. Cells were transfected with COL3A1 siRNA or control coupled with Lipofectamine® RNAi-MAX (Invitrogen) according to the product manufacture protocol, and were treated or collected at the indicated time for subsequent analysis.

### Cell cycle analysis

Primary granulosa cells treated with 5 × 10^10^ PFU/ml adv-control or adv-lnc-GULP1–2:1 (Hanbio Biotechnology, Shanghai, China) for 48 h were seeded (70% confluent) in 12-well plates for flow cytometry analysis. Cells were digested with EDTA-free trypsin, washed twice with cold PBS (1000 rpm × 5 min), and then fixed with 70% ethanol at 4 °C overnight. The fixed cells were centrifuged (1000 rpm × 5 min) again on the next day, resuspended in 500 μl of PI/RNase solution (KeyGen Biotech, Nanjing, China), and then incubated in the dark for 30 min at 37 °C. Flow cytometry studies were performed by using BD Accuri C6 Plus flow cytometer (BD Biosciences, San Jose, CA). The percentage of cells in different phases of cell cycle were analyzed by using FlowJo 10.4 (BD Biosciences).

### Statistical analysis

Data were expressed as mean ± SD. All statistical analyses were performed using SPSS 22.0 (IBM, Armonk, NY, USA). Unpaired T-test with Welch’s correction was used for the statistical analysis between the two groups, while one-way ANOVA followed by Bonferroni’s post-hoc test was used for the statistical analysis of more than two groups of data. The difference of *p* < 0.05 was considered significant. Each experiment was repeated at least three times independently.

## Results

### The expression of lnc-GULP1–2:1 in luteinized granulosa cells is correlated with ovarian function status

**Fig. 1:**
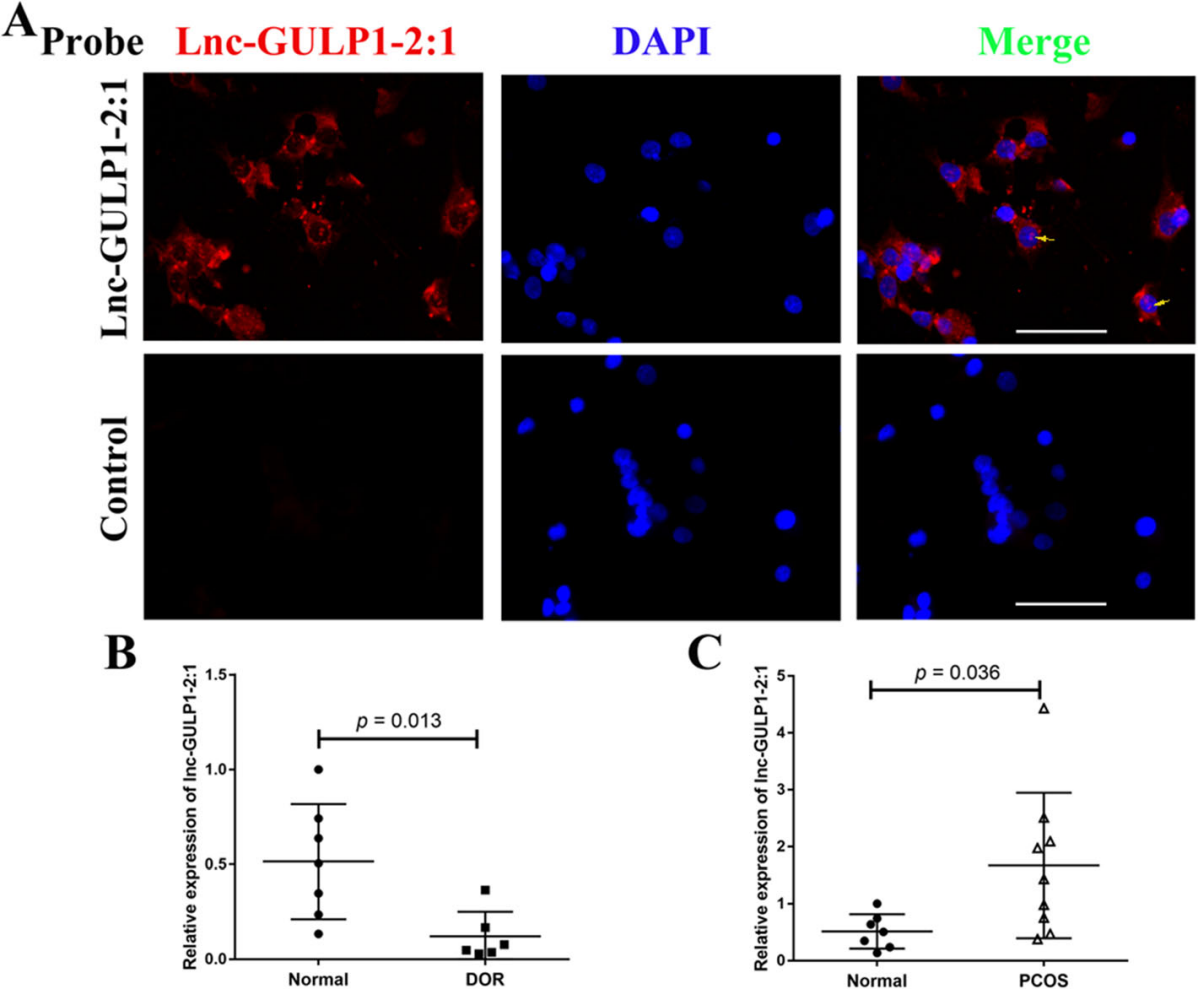
The localization and expression of lnc-GULP1–2:1 in granulosa cells. In situ hybridization was used to detect the expression of lnc-GULP1–2:1 in human primary granulosa cells (**a**). Red signal, lnc-GULP1–2:1 expression. Blue signal, DAPI signal indicates nuclear localization. The yellow arrow in the merge graph shows the punctate expression of lnc-GULP1–2:1 signal in the nucleus. Bar = 50 μm. The expression of lnc-GULP1–2:1 in granulosa cells derived from normal, DOR and PCOS patient were analyzed by using real-time PCR (**b** & **c**). The GAPDH gene was used as an internal reference. DOR, diminished ovarian reserve. PCOS, polycystic ovary syndrome. *p* < 0.05 indicates the significant difference

In this study, we focused on the role of function of lnc-GULP1–2:1 and its regulation in the regulation of ovarian follicular development. We first analyzed the localization of GULP1–2:1 in luteinized granulosa cells of IVF/ICSI patients. Immunofluorescence assay detected abundant cytoplasmic lnc-GULP1–2:1 in most of the luteinized granulosa cells. The expression signals of lnc-GULP1–2:1 can also be seen in the nucleus of some a subset of luteinized granulosa cells (Fig. 1a).

Next, we collected luteinized granulosa cells from DOR and PCOS patients to explore whether the expression of lnc-GULP1–2:1 in luteinized granulosa cells is related to different ovarian function status. The results showed that granulosa cell lnc-GULP1–2:1 expression was significantly reduced in DOR patients as compared to normal patients (*p* < 0.05) (Fig. 1b), while lnc-GULP1–2:1 levels were significantly increased in PCOS patients when compared to those in normal patients (*p* < 0.05) (Fig. 1c).

### KGN cell proliferation can be regulated by overexpression of lnc-GULP1–2:1

**Fig. 2:**
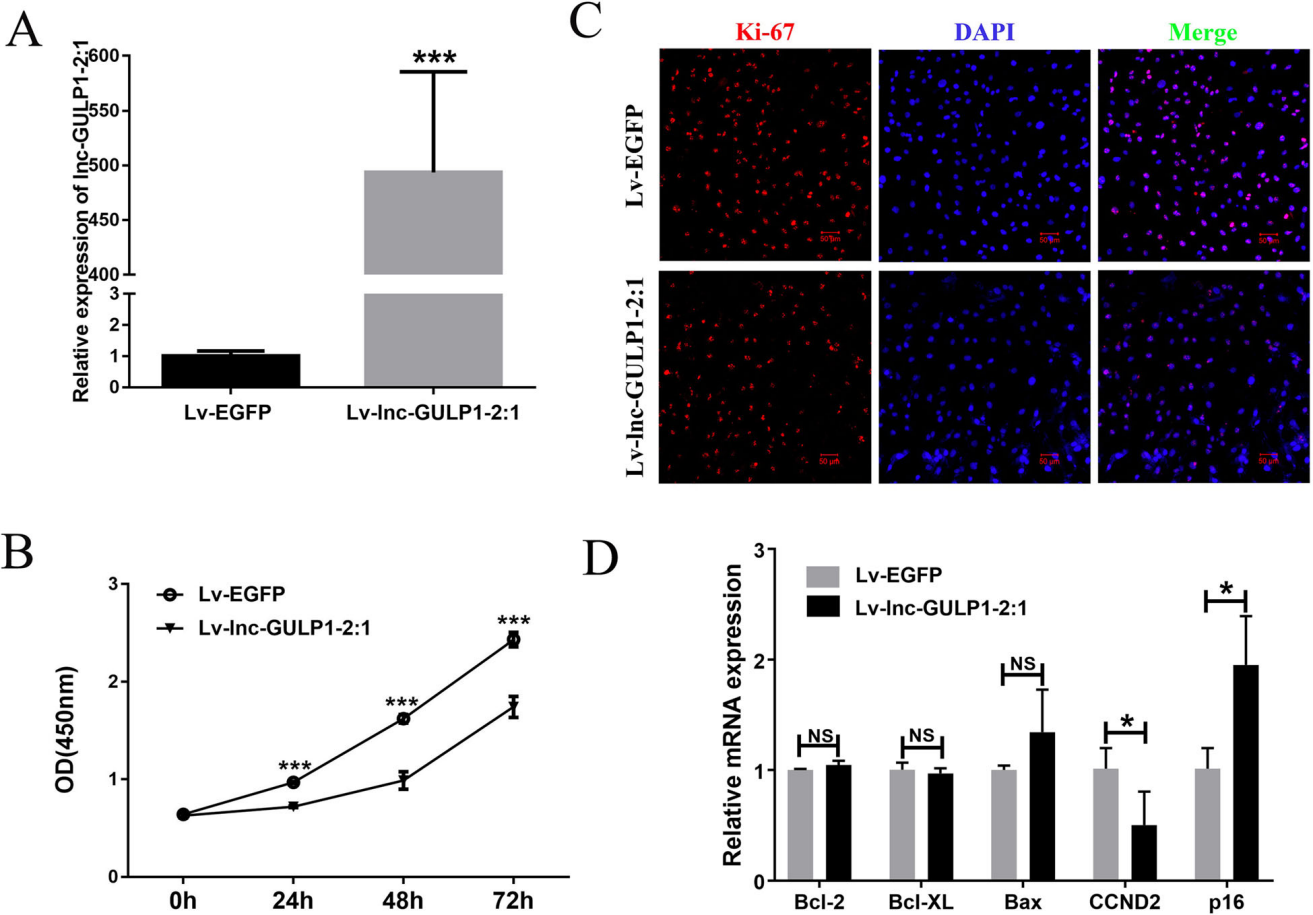
Effects of lnc-GULP1–2:1 on granulosa cell proliferation and related gene expression. Lv-lnc-GULP1–2:1 capable of overexpressing lnc-GULP1–2:1 (**a**). Cell proliferation was analyzed in group of Lv-EGFP and Lv-lnc-GULP1–2:1 at 0 h, 24 h, 48 h and 72 h by using CCK-8 (**b**). “0 h” means the beginning of cell attachment. The Ki-67 expression in KGN cell lines was analyzed by using immunofluorescence assay (**c**). Red signal, Ki-67. Blue signal, DAPI signal indicates nuclear localization. Bar = 50 μm. Real time PCR was used to detect the expression of genes related to cell proliferation, cell apoptosis and cell cycle (**d**). The GAPDH gene was used as an internal reference gene. Lv-EGFP, KGN control cell line overexpressing EGFP; Lv-lnc-GULP1–2:1, KGN cell line overexpressing lnc-GULP1–2:1. NS indicates no significant difference, * indicates *p* < 0.05, and*** indicates *p* < 0.0001

To further evaluate the regulatory role of lnc-GULP1–2:1 in granulosa cells, lentivirus-stabilized KGN cell line capable of overexpressing lnc-GULP1–2:1 (Lv-lnc-GULP1–2:1) and the control group overexpressing EGFP (Lv-EGFP) were constructed. Real-time PCR analysis showed that lnc-GULP1–2:1 overexpressed KGN cell lines were successfully constructed. We then analyzed the effect of Lv-lnc-GULP1–2:1 expression on KGN cell proliferation (Fig. 2a). Cell counting kit-8 (CCK-8) assays shows that granulosa cell proliferation was significantly inhibited in lnc-GULP1–2:1 overexpressed KGN cells (Fig. 2b). The immunofluorescence staining results of Ki-67 also showed that lnc-GULP1–2:1 inhibited the proliferation of KGN cells (Fig. 2c). Genes related to cell proliferation, apoptosis and cell cycle were also analyzed, and the results showed that overexpression of lnc-GULP1–2:1 in KGN cells had no significant effect on the expression apoptosis-inhibiting gene Bcl-2, anti-apoptotic gene Bcl-XL, and proapoptotic gene Bax (*p* > 0.05). However, cell cycle-related genes changed significantly, with the expression of cyclin D2 (CCND2) significantly reduce, while p16 increased in lnc-GULP1–2:1 overexpressed KGN cells (Fig. 2d).

### The expression and localization of COL3A1 can be regulated by lnc-GULP1–2:1 in granulosa cells

**Fig. 3:**
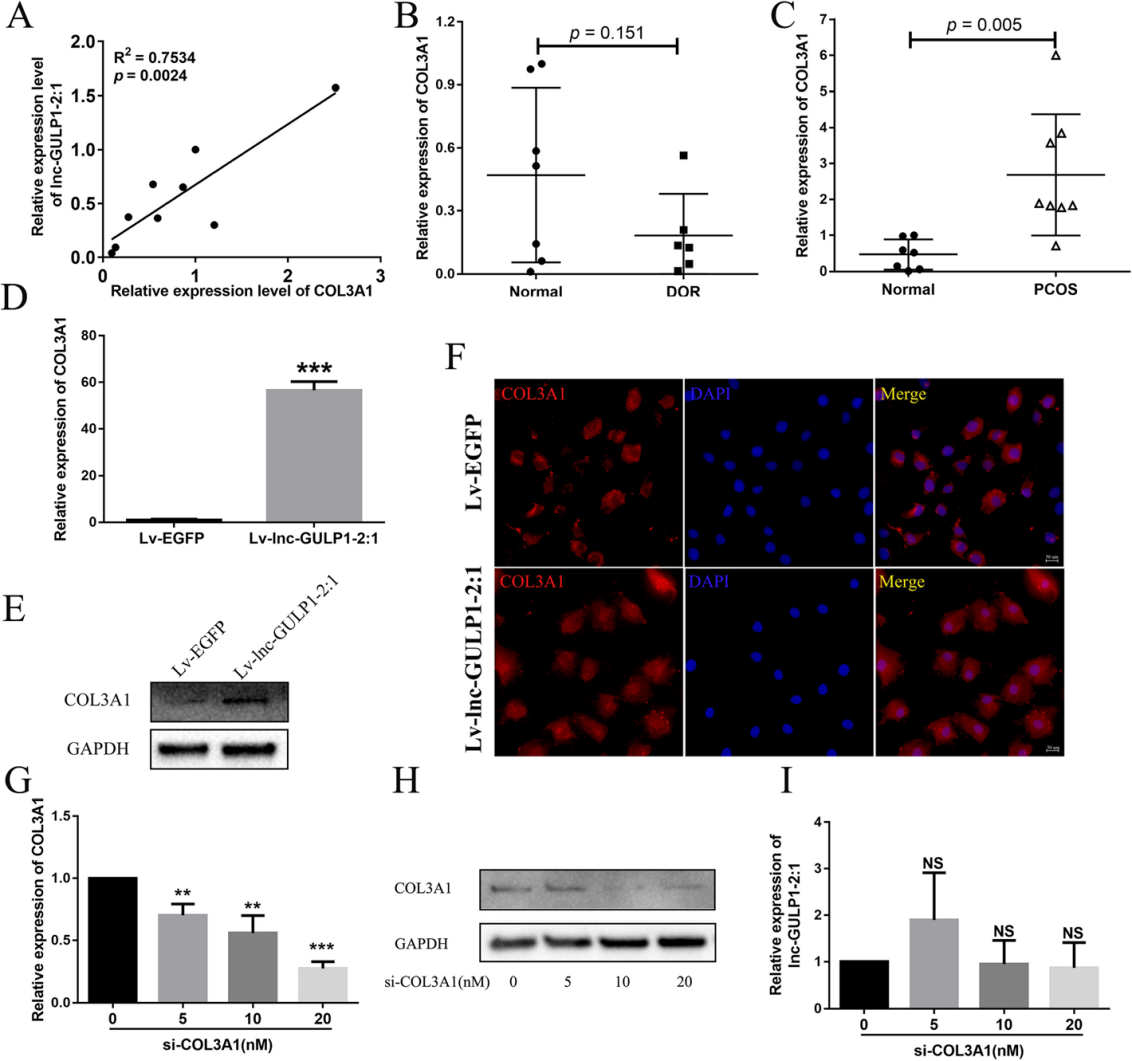
Lnc-GULP1–2:1 overexpression affects the expression and localization of COL3A1 in granulosa cells. The expression correlation of lnc-GULP1–2:1 and COL3A1 was analyzed by real-time PCR in different cell lines (including Bewo, HTR-8/SVneo, OVCAR3, SKOV3, MDA-MB-231, Hep G2, 293 T, U87 MG and JAR) (**a**). The expression of COL3A1 from patient of normal, DOR and PCOS-derived granulosa cells (**b** & **c**). The effect of overexpression of lnc-GULP1–2:1 on COL3A1 mRNA and protein expression (**d** & **e**). The expression and localization of COL3A1 in KGN cell lines analyzed by immunofluorescence assay (**f**). Red signal, COL3A1 signal. Blue signal, DAPI signal indicates nuclear localization. Bar = 50 μm. The mRNA and protein expression of COL3A1 can be dose-dependently reduced by using COL3A1 specific siRNA (**g** & **h**). Knocking down of COL3A1 on the expression of lnc-GULP1–2:1 (**i**). GAPDH was used as an internal control. NS indicates no significant difference, * indicates *p* < 0.05, ** indicates *p* < 0.01, and *** indicates *p* < 0.0001

In order to further verify the relationship between lnc-GULP1–2:1 and COL3A1, we first investigated the correlation of these two genes in different human cell lines. However, no significant correlations between the expression of lnc-GULP1–2:1 and COL3A1 were detected (*R*^2^ = 0.7534, *p* < 0.05) (Fig. 3a). In order to find out whether COL3A1 is differentially expressed in luteinized granulosa cells from patients with diverse ovarian function, we analyzed the expression of COL3A1 in luteinized granulosa cells from normal, DOR and PCOS patients. The results showed that COL3A1 expression was slightly lower in DOR patient-derived luteinized granulosa cells as compared to that in normal group (*p* > 0.05) (Fig. 3b). However, the expression of COL3A1 was significantly increased in PCOS-derived luteinized granulosa cells (*p* < 0.05) (Fig. 3c).

Consistent with our previous results, lnc-GULP1–2:1 overexpression significantly upregulated both mRNA and protein levels of COL3A1 (Fig. 3d & e). By immunofluorescence assay, we found that in Lv-EGFP control group, COL3A1 protein was mainly expressed in the cytoplasm of granulosa cells; while in Lv-lnc-GULP1–2:1 overexpression group, the expression of COL3A1 protein was increased, which was consistent with the previous western blot results. More importantly, the intracellular localization of COL3A1 also changed significantly, with COL3A1 expression in the nucleus significantly enhanced (Fig. 3f).

Lnc-GULP1–2:1 is involved in the regulation of COL3A1 expression. This together with the fact that lnc-GULP1–2:1 overlaps COL3A1 suggested that COL3A1 may regulate the expression of lnc-GULP1–2:1. By using COL3A1-specific siRNA to knockdown both the expression of COL3A1 mRNA and protein in KGN cells (Fig. 3g & h), we found there were no significant differences in the expression of lnc-GULP1–2:1, suggesting that lnc-GULP1–2:1 expression is not regulated by COL3A1 (Fig. 3i).

### KGN cell proliferation is affected by COL3A1 protein expression level and intracellular localization

**Fig. 4:**
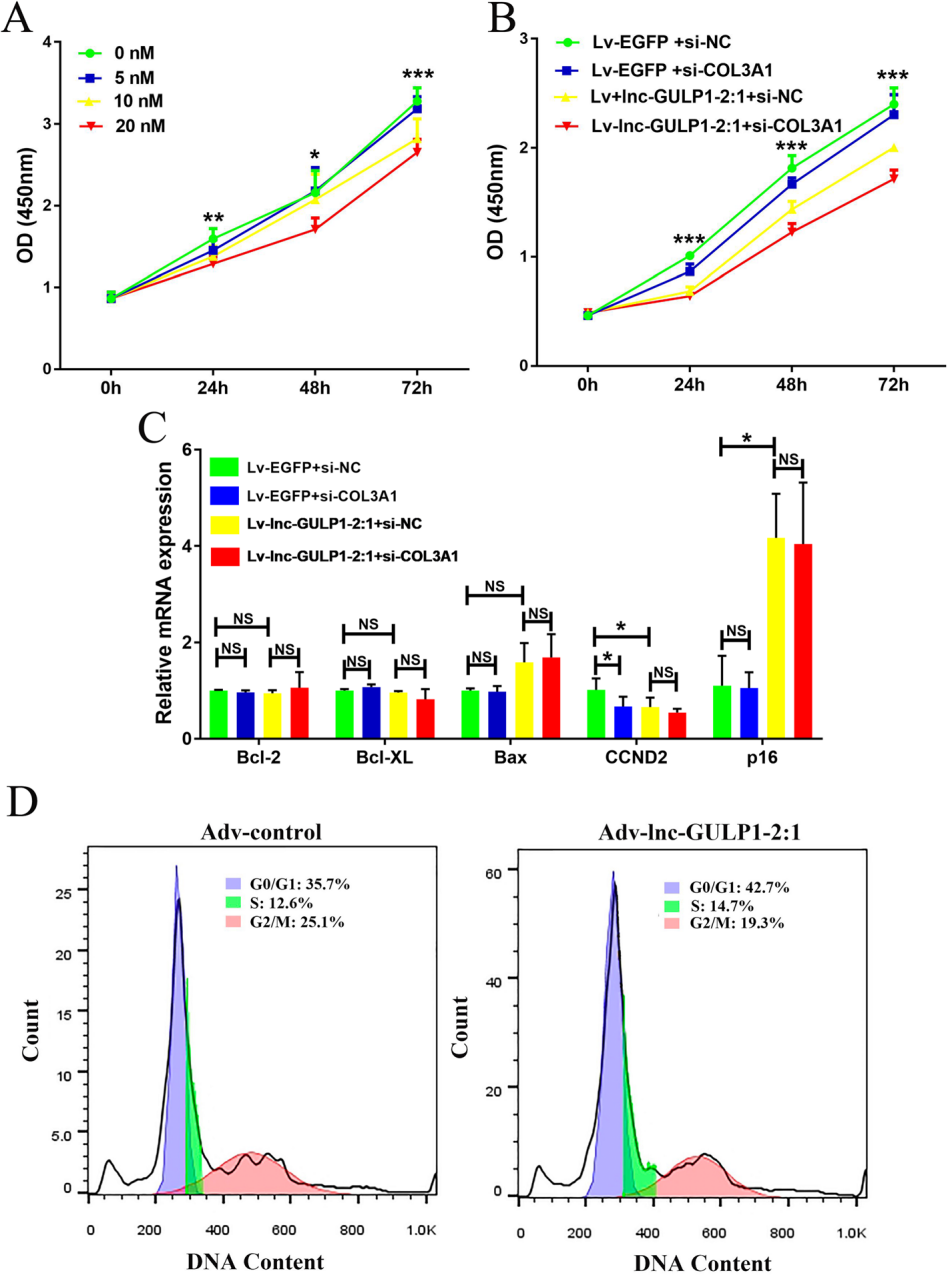
Effect of COL3A1 expression on granulosa cell proliferation and related gene expression. KGN cell proliferation was inhibited by knocking down of COL3A1 expression in dose-dependent manner (**a**). Effects of simultaneous silencing of COL3A1 expression on cell proliferation in Lv-lnc-GULP1–2:1 and Lv-EGFP cells (**b**). The expression of Bcl-2, Bcl-XL, Bax, CCND2 and p16 in Lv-lnc-GULP1–2:1 and Lv-EGFP cells by knockdown of COL3A1 (**c**). GAPDH was used as an internal control. The concentration of siRNA in plots **b** and **c** is 20 nM. NC, negative control. si-NC, silence control. NS indicates no significant difference, * indicates *p* < 0.05, ** indicates *p* < 0.01, and *** indicates *p* < 0.0001. Primary granulosa cells were infected with 5 × 10^10^ PFU/ml adenovirus overexpress lnc-GULP1–2:1 (adv-lnc-GULP1–2:1) or its control (adv-control), and flow cytometry was used to detect cell cycle changes on cells treated with adenovirus after 48 h (**d**)

Given our early date indicate that lnc-GULP1–2:1 may inhibit KGN cell proliferation by promoting COL3A1 expression, we know down COL3A1 using specific siRNAs. KGN cell proliferation is inhibited by these siRNAs in a dose-dependent manner (Fig. 4a). Furthermore, simultaneous down-regulation of COL3A1 in Lv-lnc-GULP1–2:1 and Lv-EGFP cells inhibited cell proliferation (Fig. 4b). We then analyze the role of COL3A1 in the regulation cell proliferation, apoptosis, and cell cycle. COL3A1 down-regulation did not affect the expression of Bcl-2, Bcl-XL, and Bax; However, the expression of CCND2 was significantly inhibited by COL3A1 knocking down, and further reduced in Lv-lnc-GULP1–2:1 cells. Although p16 expression was significantly increased in Lv-lnc-GULP1–2:1 cells, COL3A1 knocking down did not affect the level of p16 (Fig. 4c). In addition, we tested the effect of lnc-GULP1–2:1 on cell cycle in primary granulosa cells by infecting those cells with adenovirus overexpressing lnc-GULP1–2:1 (adv-lnc-GULP1–2:1) or its control (adv-control). The results showed that compared with the control group, lnc-GULP1–2:1 overexpression in granulosa cells increased the proportion of cells in G0/G1 phase but decreased the proportion of cells in G2/M phase (Fig. 4d). These results suggest that lnc-GULP1–2:1 increases the translocation of COL3A1 to the nucleus, thereby inhibiting granulosa cell proliferation.

## Discussion

In this study, we found that the expression of lnc-GULP1–2:1 is reduced in luteinized granulosa cells of DOR patients*.* In situ hybridization assays reveals that lnc-GULP1–2:1 is mainly localized in the cytoplasm of luteinized granulosa cell. By overexpressing lnc-GULP1–2:1 in KGN cells, we found that granulosa cell proliferation was significantly inhibited.

Both CCND2 and cyclin-dependent kinase inhibitor p16 are genes that play important roles in the regulation of cell cycle progression. By interacting with the regulatory subunits of cyclin dependent kinase 4 (CDK4) and cyclin dependent kinase 6 (CDK6), CCND2 is required for the G1 to S phase transition [27]. On the other hand, P16 inhibits cell cycle progress by slowing the G1 / S transition [28]. Lnc-GULP1–2:1 overexpression significantly enhances the expression of CCND2, but reduce the expression of p16 in granulosa cells. Furthermore, flow cytometry analysis showed lnc-GULP1–2:1 overexpression increased the proportion of G0/G1 phase cells. These results suggesting that lnc-GULP1–2:1 inhibits cell cycle progression from G1 to S phase, thereby inhibiting cell proliferation.

Lnc-GULP1–2:1 is located at the 5′ end of COL3A1 gene and overlaps partly with its transcripts. Our study showed that lnc-GULP1–2:1 affects both the expression and the localization of COL3A1. COL3A1 gene encodes the pro-alpha1 chains of type III collagen, a fibrillar collagen that is found in extensible connective tissues, an important component of the extracellular matrix [29]. Previous studies have shown that several miRNAs down-regulate the expression of COL3A1 in different disease, thus inhibiting the proliferation of corresponding cells [30–33]. Therefore, the abnormal expression of COL3A1 participate in the regulation of the proliferation and development of various cells. Other studies found that the expression of COL3A1 in cumulus cells is elevated under the stimulation of follicle stimulating hormone (FSH) [34], which can promote granulosa cell proliferation [35, 36], suggesting that COL3A1 may affect the proliferation of granulosa cells. Our study showed that COL3A1 expression levels were significantly different in granulosa cells from patients with diverse ovarian reserve. Furthermore, COL3A1 downregulation significantly inhibited granulosa cell proliferation, indicating that COL3A1 can regulate ovarian function, at least in part, by the modulation of granulosa cell proliferation.

Given the fact that granulosa cell proliferation was significantly inhibited after lnc-GULP1–2:1 overexpression, and both the expression and localization of COL3A1 can be regulated by lnc-GULP1–2:1, we propose that lnc-GULP1–2:1 may inhibit cell proliferation through the increased expression of COL3A1. However, down-regulating COL3A1 also significantly inhibited the proliferation of KGN cells. It seems that the inhibitory effect of lnc-GULP1–2:1 on KGN cell proliferation is at least partly through promoting the translocation of COL3A1 protein into the nucleus.

Though we have shown that lnc-GULP1–2:1 overexpression up-regulated the expression of COL3A1 protein and promoted the entry of COL3A1 into the nucleus, the underlying molecular mechanism is unclear. Therefore, future research is needed to show how lnc-GULP1–2:1 participates in COL3A1-mediated regulation of granulosa cell function by using bioinformatics-based lnc-GULP1–2:1 sequence mutation study, to reveal the downstream proteins regulated by lnc-GULP1–2:1 by sequencing analysis, and to explore the specific mechanism that affects the expression and localization of COL3A1.

In our study, we found that consistent with the published reports, COL3A1 knocking down decreases the cytoplasmic COL3A1 protein level, thereby affecting cell proliferation [30–33]. However, lnc-GULP-2:1 not only up-regulated the expression of COL3A1, but also promoted the entry of COL3A1 protein into the nucleus, thus decreasing cell proliferation. Therefore, both the level and subcellular localization of COL3A1 have an important influence on granulosa cell function, especially cell proliferation. Nevertheless, the mechanisms by which nucleic and cytoplasmic COL3A1 regulate cell proliferation remain to be explored.

## Conclusion

In conclusion, this study shows that lnc-GULP1–2:1 can affect the expression level of COL3A1 in cells and increase its localization in the nucleus, thereby affecting the expression level of cell cycle-associated proteins, resulting in the inhibition of granulosa cell proliferation. This study describes its regulation in granulosa cell function from the perspective of lncRNA, provides new ideas and methods for understanding and exploring the mechanism of follicular development and follicular disease, and provides a new strategy for the early diagnosis and treatment of follicular development-related diseases.

## Supplementary Information

**Additional file 1.** Sequence and chromosome localization information of Lnc-GULP1–2:1

**Additional file 2: Supplementary Table 1**. The sequences of primers used for real-time PCR

## Data Availability

The data used during this study are available from the corresponding author on reasonable request.
