# Supplementary material for: Lnc-GULP1–2:1 affects granulosa cell proliferation by regulating COL3A1 expression and localization
Source: J Ovarian Res. 2021 Jan 20;14:16. doi: 10.1186/s13048-021-00769-1 (PMC7816396; doi:10.1186/s13048-021-00769-1)
Supplement: Supplementary file 1 — Additional file 1. Sequence and chromosome localization information of Lnc-GULP1–2:1 [file 13048_2021_769_MOESM1_ESM.docx]

**Sequence and chromosome localization information of Lnc-GULP1-2:1**

**LNCipedia transcript ID:** lnc-GULP1-2:1 
**LNCipedia gene ID:** [lnc-GULP1-2](http://www.lncipedia.org/db/gene/lnc-GULP1-2)
**Location (hg19):** [chr2:189839120-189849938](http://genome.ucsc.edu/cgi-bin/hgTracks?org=human&db=hg19&position=chr2:189839120-189849938&hubUrl=http://www.lncipedia.org/trackhub/hub.txt)
**Strand:** +
**Class:** sense-overlapping
**Transcript size:** 628 bp
**Exons:** 2
**Sources:** NONCODE v4
**Alternative transcript names:** NONHSAT076037

| **NONCODE TRANSCRIPT ID** | [**NONHSAT076037.1**](http://www.noncode.org/cgi-bin/hgTracks?position=chr2:189839119-189849938&nonLnc=full&hgFind.matches=NONHSAT076037&PhyloNONCODEGene=hide&PhyloNONCODELncRNA=hide&db=hg19) |
| --- | --- |
| **Transcript desc of dated** | **Dated (reappraised by software and other databases)** |
| **NONCODE Gene ID** | [**NONHSAG030071.1**](http://www.noncode.org/show_gene.php?id=NONHSAG030071.1) |
| **Chromosome** | **chr2** |
| **Start Site** | **189839119** |
| **End Site** | **189849938** |
| **Strand** | **+** |
| **Exon Number** | **2** |
| **CNCI Score** | **-0.2679907** |
| **Length** | **628** |
| **Assembly** | **hg19** |
| **Other transcript Versions** |  |

## Sequence

**>** lnc-GULP1-2:1

**CGGTGCTGAAGGGCAGGGAACAACTTGATGGTGCTACTTTGAACTGCTTTTCTTTTCTCCTTTTTGCACAAAGAGTCTCATGTCTGATATTTAGACATGATGAGCTTTGTGCAAAAGGGGAGCTGGCTACTTCTCGCTCTGCTTCATCCCACTATTATTTTGGCACAACAGGAAGCTGTTGAAGGAGGATGTTCCCATCTTGGTCAGTCCTATGCGGATAGAGATGTCTGGAAGCCAGAACCATGCCAAATATGTGTCTGTGACTCAGGATCCGTTCTCTGCGATGACATAATATGTGACGATCAAGAATTAGACTGCCCCAACCCAGAAATTCCATTTGGAGAATGTTGTGCAGTTTGCCCACAGCCTCCAACTGCTGTGAGTTTAAAGATAAACTGTACATCTTCAATATTCATATTTAGACACATGAATAGCTCCTATATCATAGGAGCCTAAAAGGGAATGAAAGTCATGTTCATCAAATAGCCATGTTTGTATTACGAGTAAAAAGTGACCGTTTCAATTTAAAGATAAGGATTGGTTAGAATCTGGGTTACTAAATAATATGCAAATTCTGTGTCTTGTTTAACTTGTTTCTTTTCCATTTATTAGCCTACTCGCCCTCCTA**
