# Supplementary material for: Lnc-GULP1–2:1 affects granulosa cell proliferation by regulating COL3A1 expression and localization
Source: J Ovarian Res. 2021 Jan 20;14:16. doi: 10.1186/s13048-021-00769-1 (PMC7816396; doi:10.1186/s13048-021-00769-1)
Supplement: Supplementary file 2 — Additional file 2: Supplementary Table 1. The sequences of primers used for real-time PCR [file 13048_2021_769_MOESM2_ESM.docx]

**Supplementary Table 1. The sequences of primers used for real-time PCR**

| **Primer** | **Sequence of forward and reverse primers 5’-3’** |
| --- | --- |
| GAPDH | Forward Primer: 5'- ATGGAAATCCCATCACCATCTT-3' |
|  | Reverse Primer: 5'- CGCCCCACTTGATTTTGG -3' |
| lnc-GULP1-2:1 | Forward Primer: 5'- AGGATCCGTTCTCTGCGATG -3' |
|  | Reverse Primer: 5'- GACTTTCATTCCCTTTTAGGCTC -3' |
| COL3A1 | Forward Primer: 5'- AGCCTGGTAAGAATGGTGCC -3' |
|  | Reverse Primer: 5’- TCCTGGGATGCCATTTGGTC -3’ |
| Bcl-2 | Forward Primer: 5’-CATGTGTGTGGAGAGCGTCAA-3’ |
|  | Reverse Primer: 5’-GCCGGTTCAGGTACTCAGTCA-3’ |
| Bax | Forward Primer: 5'-TGCCTCAGGATGCGTCCACCAA-3' |
|  | Reverse Primer: 5'-CCCCAGTTGAAGTTGCCGTCAG-3' |
| Bcl-XL | Forward Primer: 5'- GGTCGCATTGTGGCCTTT -3' |
|  | Reverse Primer: 5'- TCCTTGTCTACGCTTTCCACG -3' |
| CCND2 | Forward Primer: 5'-TCATGACTTCATTGAGCA-3' |
|  | Reverse Primer: 5'-CACTTCCTCATCCTGCTG-3' |
| P16 | Forward Primer: 5'-ACCGAATAGTTACGGTCGGAGG-3' |
|  | Reverse Primer: 5'-CGTGGAGCAGCAGCAGCT-3' |
